# Supplementary material for: Blind spots on western blots: Assessment of common problems in western blot figures and methods reporting with recommendations to improve them
Source: PLoS Biol. 2022 Sep 12;20(9):e3001783. doi: 10.1371/journal.pbio.3001783 (PMC9518894; doi:10.1371/journal.pbio.3001783)
Supplement: S1 Fig — Original, uncropped, and unprocessed image supporting Figs 1, 2, 5 and 6. (DOCX) [file pbio.3001783.s001.docx]

**Supplementary figure 1. Unprocessed western blot related to figures 1, 2 and 5.**

**
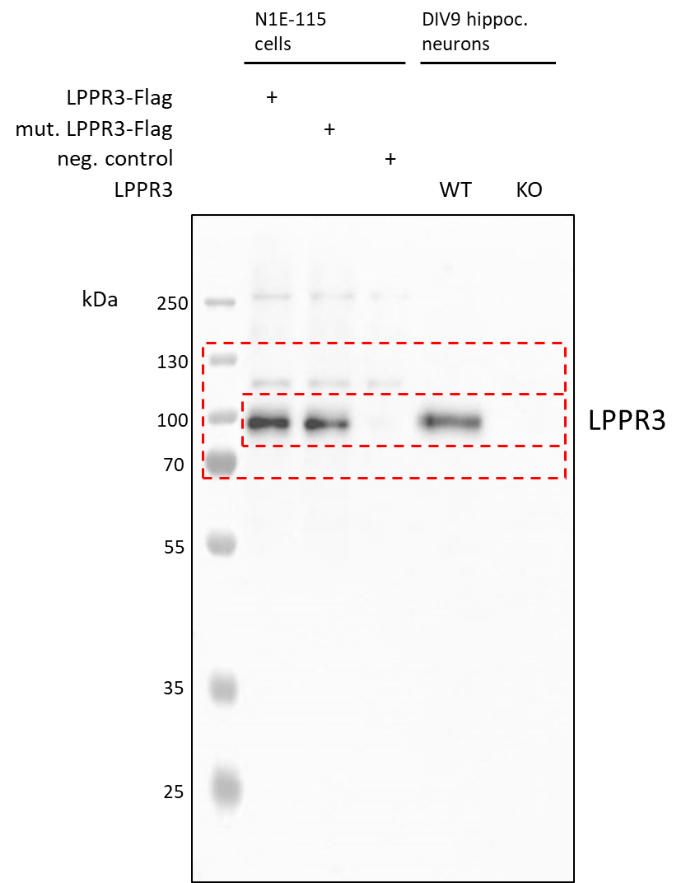
**

**Method used to capture the image:** The signal was detected with ECL Kit according to manufacturer´s instructions. The blot was incubated in 500 µl ECL reaction for 1 min and imaged using Fusion SL camera (VilberLourmat, Germany) and manufacturer´s software. The blot was imaged in auto-exposure mode with final exposure time of 3 minutes and 25 seconds. Molecular weight marker and chemiluminescent signal images were automatically overlaid by the software creating the image shown here.

For a detailed protocol of materials and methods used to make this western blot image, please see the protocol at dx.doi.org/10.17504/protocols.io.81wgb6z2olpk/v2.
