# Supplementary material for: Blind spots on western blots: Assessment of common problems in western blot figures and methods reporting with recommendations to improve them
Source: PLoS Biol. 2022 Sep 12;20(9):e3001783. doi: 10.1371/journal.pbio.3001783 (PMC9518894; doi:10.1371/journal.pbio.3001783)
Supplement: S2 Fig — This flowchart depicts the screening and selection process, including the number of journals and articles excluded and the reasons for exclusion at each stage of the study. (DOCX) [file pbio.3001783.s002.docx]

**Supplementary Figure S2: Identification of articles**

**Screening of journals**

**Identification**

Journals identified (JCR records)

Neurosciences (n = 293)

Cell Biology (n = 201)

**Journal Screening**

Journals screened (JCR records)

Neurosciences (n = 293)

Cell Biology (n = 201)

Journals excluded

Neurosciences

Not top 25% (n = 225)

No orig. research (n = 13)

Cell Biology

Not top 25% (n = 153)

No orig. research (n = 11)

Journals for which full-texts were sought for retrieval

Neurosciences (n = 55)

Cell Biology (n = 37)

Journals for which no full-texts were retrieved

Neurosciences (n = 0)

Cell Biology (n= 0)

Journals included in review

Neurosciences (n = 55)

Cell Biology (n = 37)

**Included**

**Screening of publications**

Articles excluded by automated tool

Neurosciences

No blot/gel (n = 1215)

Cell Biology

No blot/gel (n = 586)

**Article Screening**

Articles in included journals

Neurosciences (n = 1386)

Cell Biology (n = 1040)

Articles assessed for eligibility

Neurosciences (n = 171)

Cell Biology (n = 454)

Articles excluded manually

Neurosciences

Not full-length original research (n = 3)

No blot (n = 14)

No WB (n = 3)

Cell Biology

Not full-length original research (n = 31)

No blot (n = 12)

No WB (n = 11)

Articles included for abstraction

Neurosciences (n = 151)

Cell Biology (n = 400)

**Included**

Adapted from: Page MJ, McKenzie JE, Bossuyt PM, Boutron I, Hoffmann TC, Mulrow CD, et al. The PRISMA 2020 statement: an updated guideline for reporting systematic reviews. BMJ 2021;372:n71. doi: 10.1136/bmj.n71
