# Supplementary material for: Blind spots on western blots: Assessment of common problems in western blot figures and methods reporting with recommendations to improve them
Source: PLoS Biol. 2022 Sep 12;20(9):e3001783. doi: 10.1371/journal.pbio.3001783 (PMC9518894; doi:10.1371/journal.pbio.3001783)
Supplement: S1 Table — Values are n, or n (% of all articles). Articles that were not full-length original research articles (reviews, editorials, perspectives, commentaries, letters to the editor, short communications, etc.) or did not include eligible images were excluded. (DOCX) [file pbio.3001783.s003.docx]

**Table S1**

List of neuroscience journals included in the search, total number of articles screened, the number of articles identified by the tool, and the number and percentage of articles in the study.

| **Journal title** | **Total number of articles screened** | **Number of articles included by automated tool** | **Number and percentage of articles included in the study** |
| --- | --- | --- | --- |
| Acta Neuropathologica | 12 | 6 | 6 (50.00%) |
| Acta Neuropathologica Communications | 9 | 3 | 3 (33.33%) |
| Alzheimer's Research & Therapy | 14 | 1 | 0 (0.00%) |
| Annals of Neurology | 23 | 0 | 0 (0.00%) |
| Behavioral and Brain Sciences | 1 | 0 | 0 (0.00%) |
| Biological Psychiatry | 32 | 2 | 2 (6.25%) |
| Biological Psychiatry Cognitive Neuroscience and Neuroimaging | 16 | 0 | 0 (0.00%) |
| Bipolar Disorders | 11 | 0 | 0 (0.00%) |
| Brain | 38 | 2 | 2 (5.26%) |
| Brain Behavior and Immunity | 31 | 4 | 4 (12.90%) |
| Brain Pathology | 23 | 2 | 2 (8.70%) |
| Brain Stimulation | 19 | 0 | 0 (0.00%) |
| Cephalalgia | 11 | 0 | 0 (0.00%) |
| Cerebral Cortex | 59 | 7 | 5 (8.47%) |
| Cerebrovascular and Brain Metabolism Reviews | 15 | 2 | 2 (13.33%) |
| Cognitive Computation | 18 | 0 | 0 (0.00%) |
| Developmental Cognitive Neuroscience | 11 | 0 | 0 (0.00%) |
| European Journal of Neurology | 70 | 0 | 0 (0.00%) |
| Fluids and Barriers of the CNS | 6 | 0 | 0 (0.00%) |
| Frontiers in Aging Neuroscience | 102 | 9 | 8 (7.84%) |
| Frontiers in Cellular Neuroscience | 47 | 6 | 6 (12.77%) |
| Frontiers in Molecular Neuroscience | 28 | 8 | 6 (21.43%) |
| Glia | 13 | 7 | 6 (46.15%) |
| Journal of Neural Engineering | 28 | 0 | 0 (0.00%) |
| Journal of Neurochemistry | 25 | 8 | 6 (24.00%) |
| Journal of Neuroinflammation | 23 | 15 | 14 (60.87%) |
| Journal of Neuroscience | 83 | 11 | 10 (12.05%) |
| Journal of Pain | 10 | 0 | 0 (0.00%) |
| Journal of Parkinson's Disease | 35 | 3 | 2 (5.71%) |
| Journal of Pineal Research | 3 | 1 | 1 (33.33%) |
| Journal of Psychiatry and Neuroscience | 10 | 0 | 0 (0.00%) |
| Molecular Autism | 5 | 1 | 0 (0.00%) |
| Molecular Neurobiology | 37 | 21 | 21 (56.76%) |
| Molecular Neurodegeneration | 7 | 2 | 2 (28.57%) |
| Molecular Psychiatry | 38 | 7 | 7 (18.42%) |
| Multiple Sclerosis Journal | 20 | 0 | 0 (0.00%) |
| Nature Human Behaviour | 22 | 0 | 0 (0.00%) |
| Nature Neuroscience | 11 | 2 | 2 (18.18%) |
| Neural Networks | 34 | 2 | 0 (0.00%) |
| NeuroImage | 77 | 3 | 0 (0.00%) |
| Neurobiology of Disease | 14 | 4 | 4 (28.57%) |
| Neurobiology of Stress | 12 | 3 | 3 (25.00%) |
| Neurology Neuroimmunology & Neuroinflammation | 16 | 0 | 0 (0.00%) |
| Neuron | 29 | 1 | 1 (3.45%) |
| Neuropathology and Applied Neurobiology | 12 | 6 | 6 (50.00%) |
| Neuropsychopharmacology | 37 | 1 | 1 (2.70%) |
| Neuroscience & Biobehavioral Reviews | 36 | 0 | 0 (0.00%) |
| Neurotherapeutics | 20 | 8 | 8 (40.00%) |
| Pain | 38 | 2 | 2 (5.26%) |
| Progress in Neurobiology | 14 | 3 | 3 (21.43%) |
| Sleep | 33 | 0 | 0 (0.00%) |
| The Journal of Headache and Pain | 25 | 3 | 2 (8.00%) |
| Translational Neurodegeneration | 3 | 1 | 1 (33.33%) |
| Translational Stroke Research | 8 | 2 | 2 (25.00%) |
| npj Parkinson's Disease | 12 | 2 | 1 (8.33%) |
