# Supplementary material for: Blind spots on western blots: Assessment of common problems in western blot figures and methods reporting with recommendations to improve them
Source: PLoS Biol. 2022 Sep 12;20(9):e3001783. doi: 10.1371/journal.pbio.3001783 (PMC9518894; doi:10.1371/journal.pbio.3001783)
Supplement: S2 Table — Values are n, or n (% of all articles). Articles that were not full-length original research articles (reviews, editorials, perspectives, commentaries, letters to the editor, short communications, etc.) or did not include eligible images were excluded. (DOCX) [file pbio.3001783.s004.docx]

**Table S2**

List of cell biology journals included in the search, total number of articles screened, the number of articles identified by the tool, and the number and percentage of articles in the study.

| **Journal title** | **Total number of articles screened** | **Number of articles included by automated tool** | **Number and percentage of articles included in the study** |
| --- | --- | --- | --- |
| Aging Cell | 17 | 7 | 7 (41.18%) |
| American Journal of Respiratory Cell and Molecular Biology | 16 | 7 | 7 (43.75%) |
| Autophagy | 20 | 8 | 8 (40.00%) |
| Cancer Cell | 18 | 3 | 3 (16.67%) |
| Cell | 36 | 11 | 8 (22.22%) |
| Cell Calcium | 8 | 0 | 0 (0.00%) |
| Cell Death & Differentiation | 15 | 11 | 10 (66.67%) |
| Cell Death & Disease | 98 | 82 | 81 (82.65%) |
| Cell Discovery | 11 | 3 | 1 (9.09%) |
| Cell Metabolism | 16 | 3 | 3 (18.75%) |
| Cell Proliferation | 16 | 10 | 10 (62.50%) |
| Cell Reports | 134 | 66 | 56 (41.79%) |
| Cell Research | 13 | 4 | 3 (23.08%) |
| Cell Stem Cell | 8 | 1 | 1 (12.50%) |
| Cell Systems | 3 | 0 | 0 (0.00%) |
| Cellular and Molecular Life Sciences | 25 | 4 | 3 (12.00%) |
| Current Biology | 90 | 10 | 8 (8.89%) |
| Developmental Cell | 17 | 6 | 6 (35.29%) |
| EMBO Reports | 35 | 15 | 14 (40.00%) |
| Genes & Development | 11 | 6 | 6 (54.55%) |
| Journal of Biomedical Science | 5 | 4 | 4 (80.00%) |
| Journal of Cell Biology | 29 | 18 | 12 (41.38%) |
| Journal of Extracellular Vesicles | 12 | 4 | 4 (33.33%) |
| Matrix Biology | 2 | 1 | 1 (50.00%) |
| Molecular Cell | 31 | 19 | 13 (41.94%) |
| Nature Cell Biology | 18 | 6 | 5 (27.78%) |
| Nature Medicine | 38 | 3 | 0 (0.00%) |
| Nature Structural & Molecular Biology | 20 | 4 | 2 (10.00%) |
| Oncogene | 55 | 43 | 41 (74.55%) |
| Protein & Cell | 6 | 3 | 2 (33.33%) |
| Science Signaling | 12 | 7 | 6 (50.00%) |
| Science Translational Medicine | 27 | 17 | 17 (62.96%) |
| Signal Transduction and Targeted Therapy | 45 | 13 | 5 (11.11%) |
| Stem Cell Reports | 23 | 6 | 5 (21.74%) |
| Stem Cell Research & Therapy | 59 | 30 | 29 (49.15%) |
| The EMBO Journal | 23 | 13 | 13 (56.52%) |
| The Plant Cell | 28 | 6 | 6 (21.43%) |
