# Supplementary material for: Blind spots on western blots: Assessment of common problems in western blot figures and methods reporting with recommendations to improve them
Source: PLoS Biol. 2022 Sep 12;20(9):e3001783. doi: 10.1371/journal.pbio.3001783 (PMC9518894; doi:10.1371/journal.pbio.3001783)
Supplement: S3 Table — A tabular antibody reporting template. (DOCX) [file pbio.3001783.s005.docx]

**Supplementary table S3. Antibody reporting template.**

|  | **Type** | **Name** | **Source** | **Catalog nr** | **Lot nr** | **RRID** | **Dilution** |  | **Application** | **Which secondary was used (nr)?** |
| --- | --- | --- | --- | --- | --- | --- | --- | --- | --- | --- |
| 1 | Primary | Turbo-GFP | Evrogen | AB513 | 51301010912 | AB_20544089 | 1:2000 |  | WB | 3 |
|  |  |  |  |  |  |  | 1:100 |  | IHC | 5 |
| 2 | Primary | NT5D1 | Thermo Fisher Scientific | MA5-25214 | TH0923445 | AB_2723416 | 1:1000 |  | WB | 4 |
| 3 | Secondary | anti-rabbit- HRP | Innovative Research | IGAR-HRP | 150943 | AB_11041560 | 1:5000 |  | WB | NA |
| 4 | Secondary | anti-mouse- HRP | Fitzgerald Industries International | 43R-IG067hrp | 394573872 | AB_1287465 | 1:10 000 |  | WB | NA |
| 5 | Secondary | anti-mouse-Alexa488 | ThermoFisher Scientific | A97457 | W836911 | AB_2333901 | 1:500 |  | IHC | NA |
| 6 |  |  |  |  |  |  |  |  |  |  |
| 7 |  |  |  |  |  |  |  |  |  |  |
| 8 |  |  |  |  |  |  |  |  |  |  |
