# Supplementary material for: Blind spots on western blots: Assessment of common problems in western blot figures and methods reporting with recommendations to improve them
Source: PLoS Biol. 2022 Sep 12;20(9):e3001783. doi: 10.1371/journal.pbio.3001783 (PMC9518894; doi:10.1371/journal.pbio.3001783)
Supplement: Attachment — Submitted filename: v3_EditorReviewerCommentsWB.docx [file pbio.3001783.s006.docx]

**Editor Comments:**

**Editor (Comments to the Author):**

**A. Please attend to the requests from the reviewers.**

***Author Reply:*** *Our replies to the reviewers explaining how we addressed their helpful suggestions appear below.*

**B. Please change the title to something more informative and explicit; we suggest "Assessment of common problems in western blot figures and guidelines to make them informative, reproducible and transparent"**

***Author Reply:*** *Would like to keep the ‘Blind Spots in western blots’ in order to have our article appear more distinct in the sea of articles regarding this topic. We have replaced the portion of the title after the colon with a more descriptive title.*

**C. Please supply a blurb according to the instructions in the submission form.**

***Author Reply:*** *We have included a blurb in the submission system.*

**D. Please cite the location of the data clearly in the legends to Figs 3 and 4, e.g. “The data underlying this Figure can be found at https://osf.io/2c4wq/”**

***Author Reply****: We have added the suggested text and the repository link to the figure legends.*

**Reviewer Comments:**

**Reviewer #1 (Comments to the Author):**

In this manuscript, the authors carefully examined Western blots in a set of 551 articles published in top journals in the fields of neurosciences and cell biology, and scored practices such as the presence of marker lanes or marker labels, original, uncropped blots, and detailed description about blocking steps and antibodies used. Cropped blots without originals were very common, and details or labels were partially or completely missing in most of the papers. The authors provide detailed numbers on such practices, as well as guidelines to help researchers prepare figures and methods sections involving Western blots.

The manuscript is very clearly written, and the figures and textboxes are well designed and easy to understand. The article is also timely and much needed. As research papers get more complicated, details needed to replicate an experiment are often not included. Scientific journals, publishers, and peer reviewers seem to often not check if such details are included. It would be recommended if the guidelines provided in this manuscript could e.g. be included in submission guidelines or 'instructions for authors' found at scientific journals.

I enjoyed reading this manuscript and have only minor comments.

1. How did the authors define the difference between a full length blot and a cropped one? E.g. did the blot have to show edges of the membrane / film in order to be called full length, or was showing a range of e.g. 10 to 100 kDa considered full length? Some more details on the authors' definition for this study might be helpful.

***Author Reply:*** *We did not require the edges of the blot and loading lanes to be visible, as this is very rare. We defined full blots based on range of molecular weight labels, or, if no labels were present, the number of bands in the molecular weight marker. We were looking for ranges beginning in the low tens (e.g. 10-30) and going into the hundreds. The range of molecular weights shown depends on the gel percentage; therefore, it was not possible to set strict criteria. If the highest marker was closer to 100, we would expect the lowest marker to be closer to 10. If the highest marker was several hundred kDa, the lowest marker might be closer to 100. We have added the following text to the data abstraction section of the methods to clarify this point: “The edges of the gel and loading lanes were rarely shown; therefore western blots were defined as full-length based on the range of molecular weight labels or number of visible marker bands (≥ approximately 8 bands were considered sufficiently full-length). The range of molecular weights shown depends on the gel percentage; therefore, it was not possible to set strict criteria for molecular weight labels. If the highest label was closer to 100, we would expect the lowest label on a full gel to be closer to 10. If the highest label was several hundred kDa, the lowest label might be closer to 100. We are confident that all blots classified as full blots included most of the blot but cannot exclude the possibility that some very high or very low bands from the molecular weight marker were excluded.”*

**Reviewer 1**

2. General question: Did the authors look for or observe any splicing in Western blots? Splicing is another questionable research practice that might deserve to be briefly mentioned. There is a very quick mention on Page 11 ('Annotations should also note lanes that appear on the original blot but were cropped out of the region shown in the figure'). Perhaps one or two lines about splicing could be added to e.g. the discussion section. Fosang et al. can be used as a reference, since it is already used in this manuscript (reference 10).

***Author Reply:*** *We agree that splicing is an important issue, however some cases are much more obvious than others. We did not examine splicing as we did not feel that we could train reviewers to reliably detect this. We have rewritten the “Implications for future research” paragraph to mention splicing: “Finally, future studies could investigate aspects of western blot experiments that were not evaluated in this study but are critically important for reproducibility, such as procedures for antibody validation or semi-quantification of protein bands. Future studies could also assess the prevalence of questionable image preparation practices, such as splicing (reference Fosang), which were not addressed in this study.*

**Reviewer 1**

3. Page 5: Introduction: textbox 3 - no textbox 1 and 2 were mentioned before this one. Is the numbering correct? Or should this be 'textbook 1'?

***Author Reply:*** *We thank the reviewer for pointing this out. We have ensured that all textboxes are cited in order in the revised manuscript.*

**Reviewer 1**

4. Figure 3: This is minor, but perhaps relevant in a paper that discusses good practices on figure information: The figure has some inconsistencies in the labeling. In Section A, the number of papers is given in the text under the bars "% of papers (n=151)", while in Section B and C, it is given in the text above the bars "Neuroscience (n=151)".

***Author Reply:*** *We thank the reviewer for helping us to improve the figures. We have updated the figure to ensure consistent labeling by bringing the number of papers under the bar graphs in all sections of Figure 3. Unfortunately it is not possible to do the same for Figure 4, as neuroscience and cell biology share the x-axis in several panels.*

**Reviewer 1**

5. Page 8: 'Membrane blocking protocol: The membrane blocking protocol was rarely reported in detail' - I am not sure if 'rarely' is a good word here. Looking at Figure 4C, it seems that one third to half of the papers do this, so in comparison to other findings in this study, that is actually pretty good.

***Author Reply:*** *We agree that “rarely” is misleading here. We have changed the text “Details of the membrane blocking protocol were often missing.”*

**Reviewer 1**

6. Page 9: What exactly did the authors mean by papers reporting on the source of primary antibodies? Does it refer to the name of the manufacturer, or the animal species in which it was generated? The word 'source' is also used in other parts of this manuscript to describe original, uncropped blots, so it would be good to give a precise definition of the word 'source' in this particular context here.

***Author Reply:*** *We thank the reviewer for helping us to clarify the manuscript. We have replaced the term “source” with “Company or supplier” in Figure 4D and in the text.*

**Reviewer 1**

7. Page 9, box "Reporting Replicates". For biological replicates, the authors write "e.g. 5 patients or mice" but I am not sure if samples from 5 patients could be considered biological replicates, since they are from different individuals. Rather, one might think of e.g. 5 blood or stool samples from the same patient, or even different aliquots from the same stool sample. For inbred mice kept at the same conditions, one might argue that these are biological replicates, but humans have much more variation. See e.g. https://www.nature.com/articles/nmeth.3091 But I will admit that I am not sure about the definition, and I will leave it up to the authors to decide if this wording can stay.

**Author Reply:** *Scientists often misinterpret or incorrectly use terms like biological replicate, technical replicate and pseudoreplicate. Biological replicates do, in fact, refer to independent samples – this is confusing for many scientists because they don’t consider independent samples to be replicates. We have adapted the first paragraph of textbox 1 to more clearly address some of the confusion surrounding these terms, and recommend that authors clearly describe what they did rather than relying on these problematic terms. The first paragraph of textbox 1 now reads as follows:*

*“What causes the confusion in reporting experimental replicates?
Replicates provide evidence that experimental results are reproducible. Reporting the number and type of replicates increases transparency and trustworthiness and allows the readers to assess the generalizability of the findings. Unfortunately, in our experience, the terms used to describe replicates are interpreted differently by researchers in different fields and are often used incorrectly. Genuine replicates, which are sometimes called biological replicates, are independent, provide information about variability between samples and increase the sample size (Lazic 2018). An example of this would be measuring the level of a protein of interest in the brain lysates of 5 mice. These terms can be confusing, as many scientists do not consider independent samples to be replicates. Pseudoreplicates, which are sometimes called technical replicates, are not independent and do not increase sample size (Lazic 2018). They only provide information about measurement errors. An example of this would be measuring the level of a protein of interest in the brain lysate of one mouse 5 times. These terms can also be confusing, as the degree of non-independence required to define something as a technical replicate may differ among labs and fields. Given the potential for misinterpretation and misuse, we encourage authors to clearly describe what they did and avoid using terms like biological replicates, technical replicates and pseudoreplicates. Authors should concisely explain what samples were measured, and if and how samples were related to each other (e.g. independent, matched, samples from different tissues in the same animal, etc.).”*

**Reviewer 1**

8. Page 9, box "Reporting Replicates": it might be better to change the word "source" in "Technical replicates are not independent as these samples originate from the same source" to "specimen", since the word 'source' is also used to describe other things in this study.

***Author Reply:*** *Thank you for this suggestion. The word “source” was eliminated while editing this section, as described in the previous point.*

**Reviewer 1**

9. Page 10. "Some journals and guidelines [11, 12, 23] require authors to submit original uncropped images of blots". Were any of the journals included in this study journals that required authors to submit uncropped blots (e.g. in the supplemental materials)? If so, did this study still find that such originals were missing? As a suggestion for an additional analysis on this dataset, the authors could add such findings to e.g. Figure 3A, or they could make a brief statement about their findings. Did those journals requiring original blots do better in the analysis here? This additional analysis is just a suggestion, not a requirement on my behalf for acceptance of this manuscript.

***Author Reply:*** *Thanks to the reviewer's interest, it has come to our attention that we had some inconsistencies in our references. We have changed the references to now cite British Journal of Pharmacology and PLOS Biology as journals that require authors to submit source data for peer review, and Nature group as journals that explicitly state that source data will be published. As to the suggestion of the reviewer, we would have loved to analyze whether source data was more often present in those journals. However, considering the small number of papers that included blot source data, a journal-wise analysis would not yield meaningful data.*

**Reviewer 1**

10. Page 12, Figure 5. "Published western blots should show a minimum of two molecular weight markers" - maybe word this a bit more precisely. The authors probably do not mean a minimum of two lanes with markers, but a minimum of two bands of different weight.

***Author Reply:*** *Thank you for this suggestion. We have adjusted the sentence as follows: “Published western blots should show a minimum of two molecular weight markers bands of different weights if the protein of interest falls between the molecular weight marker bands, and three molecular weight marker bands of different weights if the protein of interest is directly at one of the marker bands.”*


**Reviewer #2 (Comments to the Author):**

The authors inspected Western blots in 551 original research articles across 92 journals, 55 from neurosciences and 37 from cell biology. The top 25% of journals from each category were selected.
* most published Western blots are cropped and blot source data are not made available to readers in the supplement.
* publishing blots with visible molecular weight markers is rare, and many blots additionally lack molecular weight labels.
* Western blot methods sections often lack information on the amount of protein loaded on the gel, blocking steps and antibody labeling protocol.
* Important antibody identifiers like source, catalog number or RRID were omitted frequently for primary antibodies, and regularly for secondary antibodies.
Their data reveal a significant lack of attention by journals in this regard, and points to a need for improved reporting standards, and more specific recommendations in journal guidelines.
 The article is well written and the figures are clear and informative. The authors also include a toolbox to help scientists produce more reproducible western blot data, teaching slides in English and Spanish, and an antibody reporting template.
The authors describe common shortcomings in methods reporting, data visualization, and image display. Addressing these would significantly improve assessability, reproducibility and trustworthiness of the research reported.

Suggestions:

**Reviewer 2**
 * The authors mention that proper reporting 'may come at a cost ' in the Introduction, but do not discuss this much further. The cost is presumably mainly monetary (e.g. cutting blots to save antibodies), although there may also be a time factor. It appears that blotting techniques differ around the world, as do standards of imaging and how the raw images are prepared and stored. Geographic differences are not mentioned or discussed.

***Author reply:*** *The reviewer refers to the following sentences, which describes the benefits of digital cropping but notes that there are significant disadvantages to this common approach: “”Digital cropping to display only the bands of interest can save space, allow authors to efficiently combine many blots into a single figure, and focus readers’ attention on the band of interest. However, these benefits come at a cost.” We have replaced “come at a cost” to “deprive readers of essential information” to be more specific.*

*We have added new text to address the issue of the cost of implementing more transparent practices in the "How can authors make their western blot images more informative?” section, as follows: “In some laboratories, researchers may physically cut membranes prior to antibody incubation. Some laboratories may do this out of habit, whereas others may want to conserve expensive antibodies. In addition to making it impossible to detect additional bands, this practice also makes it difficult to confirm that controls were run on the same gel. The requirement to provide full blots for every blot relevant to the paper would place labs with limited resources, including those in countries with limited research funding, at a disadvantage. As a compromise, authors could show at least one full-length replicate of each individual western blot assay.”
We have also addressed this in the “Implications for journal policies” section: “While there are clear advantages to requiring full blots, doing so would place labs with limited resources at a disadvantage. As a compromise, journals could ask authors to provide at least one full-length blot for each western blot assay.”*

**Reviewer 2**
 * The authors briefly mention image integrity. They could point out that full information and complete raw data are crucial during investigations addressing questions regarding potential misconduct issues or errors (experimental or during figure preparation). Journals should routinely request to see the original and full raw Western blot images prior to publication, even if they aren't published alongside the research articles in the end.

***Author reply:*** *We thank the reviewer for this comment and agree that a stronger recommendation for the journals is suitable. We have included the following sentences in the “Implications for journal policies” section: “All journals should routinely request full-length western blot source data prior to publication to ensure these data exist (ref 25, Miyakawa). Ideally, these data should be available to readers once the paper is published. Source data can help editors, reviewers and authors to address questions about potential errors or misconduct (e.g. duplicated images) (reference* [*https://doi.org/10.1128/mBio.00809-16*](https://doi.org/10.1128/mBio.00809-16)*). While there are clear advantages to requiring full blots, doing so would place labs with limited resources at a disadvantage. As a compromise, journals could ask authors to provide at least one full-length blot for each experimental assay.”*

**Reviewer 2**
* Over-exposure or over-contrasting of the final images, resulting in a lack of background noise is not mentioned although this is a common problem. Over-contrasting can be used to hide weaker signals/bands, to hide splice marks and cuts as well as a variety of other digital image manipulation issues.

***Author reply:*** *We thank the reviewer for this comment and agree that presenting blots with white background is a major issue that can also conceal many other manipulations. We’ve added a new section to the discussion highlighting some practices that we did not collect data on, including white background and splicing (mentioned by Reviewer 1). We also added Figure 6 to illustrate the white background issue. This section also refers readers to Textbox 3, which has additional resources covering many other aspects of improving western blots.*

*Note that we refer to the lack of background issue described by the reviewer as white background, as the terms over-exposure and over-contrasting might be confusing to some and blur the lines between appropriate and inappropriate manipulation. We would define over-exposure as an excessive duration of exposure leading to very dark blots or blown out bands. Over-contrasting to us implies the inappropriate use of non-uniform contrast adjustments, which lighten light sections of the blot and darken dark sections of the blot. This is quite different from uniform lightening of the blot, which is an acceptable adjustment.*

*The text of the new section is as follows:*

*Additional strategies and resources for improving western blots*

*While this paper addressed several practices related to the display of western blot images, data presentation and methods reporting, there are many more important practices that were beyond the scope of the current study. Proper labeling of spliced blots, for example, is critically important to show readers how the blot presented in the paper differs from the source image (ref 10). It’s also important to ensure that the blot background is gray, rather than white. While scientists are permitted to lighten or darken blots, these adjustments should be uniform across the entire blot (ref 43). Once any section of the blot background is white, or maximally lightened, any further lightening would be non-uniform. Excessive lightening can be used to reduce background (Figure 6, panel B) and, in highly problematic cases, to eliminate faint bands (Figure 6, panel C) or conceal other potentially problematic adjustments like improper splicing. Presenting blots with a light gray background that is clearly distinguishable from the white page allows readers to confirm that excessive lightening was not performed. Textbox 3 provides an overview of additional resources to help scientists improve western blot methods and reporting, including aspects not discussed in this paper.*

**Reviewer 2**
 * Regarding cropping: It might be worth explaining that in some countries it appears to be commonplace to cut membranes into slim strips before they are placed into an imaging device. Instead of blots displaying the entire vertical length of the gel, these strips are often the only raw data available and typically just show the band of interest, frequently also without showing molecular weight markers and -labels. This also makes it difficult to verify whether controls were run on the same blot as the proteins of interest. If confronted, authors often explain that this is common practice to cut costs. Some journals appear to still accept this type of raw data despite the clear drawbacks.

***Author reply:*** *We fully agree with the reviewer about the challenges that cutting blots poses to the integrity of experimental data. This is a challenging issue to address. Full blots are much better for allowing others to verify the experimental results; yet requiring full blots for every experiment and replicate places labs with limited resources, regardless of geographic location, at a disadvantage. We have amended the text as follows to acknowledge the complexity of this issue.*

*“In some laboratories, researchers may physically cut membranes prior to antibody incubation. Some laboratories may do this out of habit, whereas others may want to conserve expensive antibodies. In addition to making it impossible to detect additional bands, this practice also makes it difficult to confirm that controls were run on the same gel. The requirement to provide full blots for every blot relevant to the paper would place labs with limited resources, including those in countries with limited research funding, at a disadvantage. As a compromise, authors could show at least one full-length replicate of each individual western blot assay.”*
*A respective recommendation was also included in the “Implications for journal policies” section: “As a compromise, journals could ask authors to provide at least one full-length blot for each experimental assay.”*

**Reviewer 2**
The results of the present study reveal a lack of fastidiousness by some journals and could be an important contribution enforcing improved reporting standards.

**Author Reply:**

*We thank the reviewer for this comment and hope that the paper will help authors, reviewers and editors to improve the quality of published western blot images, data and methods.*
